# Old-Age and Eyesight

**Published:** 1914-06-13

**Authors:** 


### Old-Age and Eyesight.

An interesting, though inconclusive, correspondence has been going on in the *Times* this week on the subject of presbyopia, that failure of the accommodative power of the eye for near vision which compels most middle-aged and elderly people to resort to spectacles for reading, sewing, and similar occupations. A correspondent who signed himself "F.R.C.S." (and described himself as an ophthalmic surgeon) wrote to point out that when centenarians or other very old people are able to read and write without spectacles, proof is thereby given not that their eyesight is exceptionally perfect, but merely that they suffered in earlier life from short sight. This is, medically speaking, a truism well known to the profession, though not appreciated by the public. The enunciation of this fact sufficed to elicit semi-indignant answers from several octogenarians, who all protested their ability to read without glasses and their possession of normal eyesight in their younger days. Nevertheless, the veterans are quite in the wrong, however difficult it may be to persuade them so; and "F.R.C.S." is perfectly right. Where their evidence fails to carry conviction is in regard to their eyesight in youth. It is quite easy to be shortsighted without being oneself aware of it; and it was easier still when those who are now of eighty years or more were in their prime, for ophthalmic surgery was in its infancy, and only a small proportion of those with defective vision ever received attention for it.

